# The prognosis of *MYC* translocation positive diffuse large B‐cell lymphoma depends on the second hit

**DOI:** 10.1002/cjp2.10

**Published:** 2015-03-30

**Authors:** Alexandra Clipson, Sharon Barrans, Naiyan Zeng, Simon Crouch, Nicholas F Grigoropoulos, Hongxiang Liu, Sylvia Kocialkowski, Ming Wang, Yuanxue Huang, Lisa Worrillow, John Goodlad, Jenny Buxton, Michael Neat, Paul Fields, Bridget Wilkins, John W Grant, Penny Wright, Hesham EI‐Daly, George A Follows, Eve Roman, A James Watkins, Peter W M Johnson, Andrew Jack, Ming‐Qing Du

**Affiliations:** ^1^ Division of Molecular HistopathologyDepartment of PathologyUniversity of CambridgeUK; ^2^ Haematological Malignancy Diagnostic ServiceSt. James's Institute of OncologyLeedsUK; ^3^ Department of Health SciencesEpidemiology and Cancer Statistics GroupUniversity of YorkYorkUK; ^4^ Department of HaematologyAddenbrooke's Hospital, Cambridge University Hospitals NHS Foundation TrustCambridgeUK; ^5^ Department of HistopathologyAddenbrooke's Hospital, Cambridge University Hospitals NHS Foundation TrustCambridgeUK; ^6^ Department of PathologyWestern General HospitalEdinburghUK; ^7^ Department of HaematologyWestern General HospitalEdinburghUK; ^8^ Department of Haematology and Department of CytogeneticsGSTS PathologyGuy's and St. Thomas NHS Foundation TrustLondonUK; ^9^ Department of Haematology GSSTKings Health PartnersLondonUK; ^10^ Histopathology DepartmentSt Thomas' Hospital and King's CollegeLondonUK; ^11^ Cancer Research UK CentreUniversity of SouthamptonSouthamptonUnited Kingdom

**Keywords:** DLBCL, chromosome translocation, TP53 mutation, double‐hit, overall survival

## Abstract

A proportion of *MYC* translocation positive diffuse large B‐cell lymphomas (DLBCL) harbour a *BCL2* and/or *BCL6* translocation, known as double‐hit DLBCL, and are clinically aggressive. It is unknown whether there are other genetic abnormalities that cooperate with *MYC* translocation and form double‐hit DLBCL, and whether there is a difference in clinical outcome between the double‐hit DLBCL and those with an isolated *MYC* translocation. We investigated *TP53* gene mutations along with *BCL2* and *BCL6* translocations in a total of 234 cases of DLBCL, including 81 with *MYC* translocation. *TP53* mutations were investigated by PCR and sequencing, while *BCL2* and *BCL6* translocation was studied by interphase fluorescence in situ hybridization. The majority of *MYC* translocation positive DLBCLs (60/81 = 74%) had at least one additional genetic hit. In *MYC* translocation positive DLBCL treated by R‐CHOP (*n* = 67), *TP53* mutation and *BCL2,* but not *BCL6* translocation had an adverse effect on patient overall survival. In comparison with DLBCL with an isolated *MYC* translocation, cases with *MYC/TP53* double‐hits had the worst overall survival, followed by those with *MYC/BCL2* double‐hits. In *MYC* translocation negative DLBCL treated by R‐CHOP (*n* = 101), *TP53* mutation, *BCL2* and *BCL6* translocation had no impact on patient survival. The prognosis of *MYC* translocation positive DLBCL critically depends on the second hit, with *TP53* mutations and *BCL2* translocation contributing to an adverse prognosis. It is pivotal to investigate both *TP53* mutations and *BCL2* translocations in *MYC* translocation positive DLBCL, and to distinguish double‐hit DLBCLs from those with an isolated *MYC* translocation.

## Introduction

Diffuse large B‐cell lymphoma (DLBCL) represents 30–45% of adult cases of non‐Hodgkin lymphoma (https://www.hmrn.org/statistics) 1, accounting for more than 80% of aggressive lymphomas. The addition of rituximab has significantly improved the treatment outcome of patients with DLBCL. Nonetheless, a significant proportion of DLBCLs show primary treatment failure (∼10%), partial response (∼15%) or relapse after initial response (20–30%) to R‐CHOP (rituximab, cyclophosphamide, doxorubicin, vincristine and prednisone), the current first line treatment for this malignancy 2. A number of biomarkers have been investigated with the aim of predicting treatment outcome at diagnosis and identifying those that may benefit from novel therapeutic strategies, but only a few have proven to be clinically useful due to lack of reproducibility (immunohistochemistry‐based markers) and/or difficulty of routine clinical application (molecular subtypes by gene expression profiling) on formalin‐fixed paraffin‐embedded diagnostic tissue biopsies 3, 4, 5. Among the many biomarkers investigated, *MYC* chromosome translocation is widely accepted and used in routine clinical practice.

The *MYC* translocation occurs in 5–15% of DLBCL, and is usually associated with a complex pattern of genomic alterations 6, 7, 8, 9, 10, 11, 12. A proportion (21–83%) of DLBCLs with *MYC* translocation also harbour a *BCL2* and/or *BCL6* translocation, known as ‘double‐hit’ or ‘triple‐hit’ lymphoma. Patients with ‘double‐hit’ DLBCL commonly show aggressive clinical features and respond poorly to currently available treatments, with a median survival less than 1.5 years 6. However, it remains controversial whether DLBCL with *MYC* single translocation has a different prognosis from that with *MYC*/*BCL2* double translocation. For example, the recent studies by Cuccuini *et al* 13, Aukema *et al* 6 and Valera *et al* 14 showed a similar poor overall survival between DLBCL with *MYC* single translocation and those with *MYC* double translocations. In contrast, the studies by Johnson *et al*, Green *et al* and Landsburg *et al* demonstrated no adverse impact of *MYC* single translocation in DLBCL, while cases with *MYC/BCL2* double translocations had a very poor outcome 15, 16, 17. Although the reasons underlying the discrepancies between these studies are unclear, potential factors that may account for the discrepancies could include the small numbers of cases investigated, variations in clinicopathological parameters (age, stage, international prognostic index [IPI]) and a variable presence of additional genetic changes such as *TP53* mutation that modifies the prognostic value of *MYC* translocation.

MYC drives cell proliferation but also sensitizes cells to apoptotic stimuli, which provides a safeguard to prevent any potential MYC induced malignant transformation. The MYC mediated proapoptotic activity is largely through the activation of the p19(ARF)‐MDM2‐TP53 pathway and repression of the apoptosis inhibitor BCL2 18, 19. There is extensive literature showing that MYC requires cooperating events to abrogate its proapoptotic activities to exert its full oncogenic potential, and both expression of *BCL2* and loss of *TP53* function cooperate with *MYC* translocation in lymphomagenesis 18, 19, 20, 21. In DLBCL, *TP53* mutations are found in ∼20% of cases and are significantly and independently associated with poor overall survival of both activated B‐cell like (ABC) and germinal centre B‐cell like (GCB) DLBCL treated with R‐CHOP 22, 23. *TP53* mutations have been reported in cases of DLBCL with *MYC* translocation 24, 25, but their frequency in *MYC* translocation positive DLBCL and their combined clinical impact are unclear. In this study, we have investigated *TP53* mutations and *BCL2* translocations in a large cohort of DLBCL, and analysed their association and clinical impact in the presence and absence of *MYC* translocation.

## Materials and Methods

### Patients and tissue materials

A total of 234 cases of de novo DLBCL were investigated in this study. 168 cases were retrieved from the Haematological Malignancy Diagnostic Service (HMDS) at St James's University Hospital, Leeds (*n* = 145) and Addenbrooke's hospital, Cambridge (*n* = 23), based on the availability of lymphoma tissue specimens. 153 of these cases have been classified by cell of origin (COO) using the Illumina WG‐DASL assay used in previous studies 7, 26. The remaining 66 cases were positive for *MYC* translocation by interphase fluorescence in situ hybridisation (FISH) and identified from five participating centres where the assessment of *MYC* translocation status is a part of the routine diagnostic workup of DLBCL 27. The diagnosis in each case was established by two expert haematopathologists, and those defined as B‐cell lymphoma, unclassifiable, with features intermediate between DLBCL and Burkitt lymphoma, in the 2008 WHO classification were included in this study 28. Burkitt lymphoma, DLBCL potentially transformed from a low grade lymphoma, cases with HIV or primary CNS lymphoma were excluded from this study. All the laboratory investigations described below were based on the initial diagnostic lymphoma tissue specimens. Ethical guidelines were followed for the use of archival tissues for research with the approval of the ethics committees of the involved institutions.

### Microdissection and DNA preparation

Haematoxylin and eosin slides were reviewed for all cases, and crude microdissection was performed where indicated to enrich tumour cells, ensuring that the tissue area containing >60% of tumour cells was used for DNA preparation. DNA was extracted using the QIAamp DNA Micro Kit (QIAGEN, Crawley, UK). The quality of the DNA samples was assessed by polymerase chain reaction (PCR) of variably sized genomic fragments 29, and those with successful amplifications of >300 bp were used for mutational screening.

### PCR and Sanger sequencing

Mutations in *TP53* coding exons 5–10, commonly targeted by somatic mutation in human cancer, were investigated by PCR and Sanger sequencing using primers and conditions detailed in supplementary material Table S1. In each case, sequence change was confirmed by at least two independent PCR and sequencing experiments. The somatic mutation was ascertained by excluding germline changes through SNP database search and analysis of DNA prepared from microdissected normal cells, where possible.

### Interphase FISH

Chromosome translocations involving the *MYC*, *BCL2* and *BCL6* loci were investigated using dual‐colour break‐apart probes (Vysis/Abbott Laboratories, UK) 30. For each probe, the mean plus three standard deviations of false positive signals in 100 nuclei from 8 to 10 reactive tonsils was used as the cut‐off value for the diagnosis of chromosomal translocation. The cut‐off value for *MYC*, *BCL2* and *BCL6* break‐apart probes is very similar, all being <6%.

### Statistical analyses

Rates of genetic alterations and molecular subtypes by COO classification were compared using difference‐of‐proportions tests. Survival analyses were performed using the Kaplan–Meier method with log‐rank tests and by using the Cox proportional hazards model.

## Results

We investigated a total of 234 cases of primary DLBCL, including 168 cases selected based on their tissue availability, and 66 additional cases based on their positivity for *MYC* translocation. Among the 168 cases selected based on tissue availability, the frequencies of *MYC* (15/168 = 8.9%), *BCL2* (30/168 = 17.8%) and *BCL6* (49/167 = 29.3%) translocation and *TP53* mutation (26/166 = 15.7%) were in line with those reported in the literature 6, 22, 23, 31, 32. To understand the potential oncogenic cooperation of these genetic changes, we analysed associations across the entire patient group.

### High frequencies of *TP53* mutation and *BCL2* translocation in *MYC* translocation positive DLBCL

**Figure 1:**
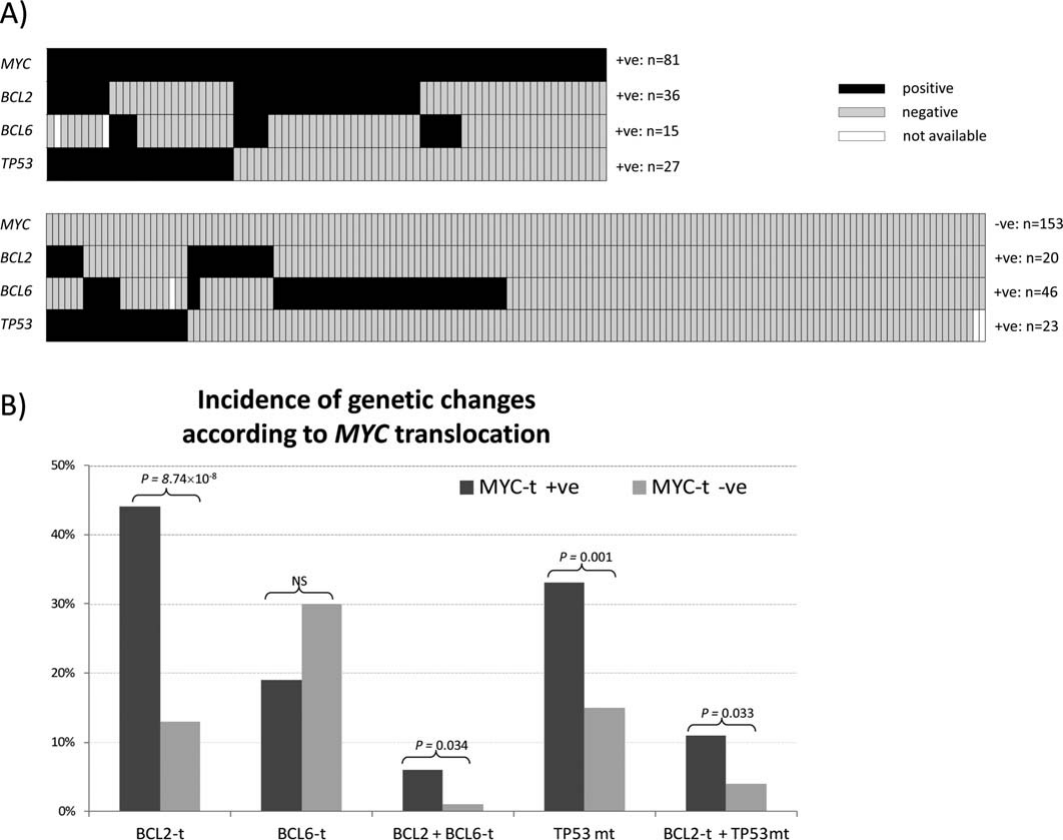
Correlation of *TP53* mutation, *MYC*, *BCL2* and *BCL6* translocation in primary DLBCL. (A) Distribution of *TP53* mutation, *BCL2* and *BCL6* translocation in DLBCLs with and without *MYC* translocation. The majority of *MYC* translocation positive DLBCLs harbour at least one additional genetic abnormality, frequently *TP53* mutation or *BCL2* translocation, and occasionally *BCL2* translocation plus *TP53* mutation or *BCL6* translocation. Black cell: positive for the genetic abnormality indicated; Grey cell: negative for the genetic abnormality indicated; White cell: data not available. (B) Incidence of *TP53* mutation, *BCL2* and *BCL6* translocation in DLBCLs with and without *MYC* translocation. The frequency of *TP53* mutation and *BCL2* translocation is significantly higher in cases with *MYC* translocation. t: translocation; +ve: positive; ‐ve: negative; NS: no significance.

The frequency of *TP53* mutation was significantly higher in cases with *MYC* translocation than in those without the translocation (27/81 = 33.3% versus 23/151 = 15.2%, *p =* 0.001, Figure 1A,B). However, there was no significant difference in the frequency of *TP53* mutation between DLBCL with an isolated *MYC* translocation and those with double translocations (*MYC* plus *BCL2* or *BCL6*) (14/35 = 40% versus 13/46 = 28.3%, *p =* 0.38). Five cases (6% within the *MYC* translocation positive series) were found to have concurrent translocations of *MYC*, *BCL2* and *BCL6*, and none of these had a *TP53* mutation (Figure 1A). In addition, no differences in the spectrum of *TP53* mutations were observed between DLBCL with *MYC* translocation and those without (supplementary material Figure S1).

The frequency of *BCL2* translocation was also significantly higher in DLBCL with *MYC* translocation than in those without (36/81 = 44.4% versus 20/153 = 13.1%, *p* = 8.74 × 10^−8^, Figure 1B). In contrast, the frequency of *BCL6* translocation was lower in DLBCL with *MYC* translocation than in those without (15/79 = 18.9% versus 46/152 = 30.0%, *p* = 0.065, Figure 1B).

In cases with *MYC* translocation, there was no evidence of correlation among *TP53* mutation, *BCL2* and *BCL6* translocation (supplementary material Table S2). In cases without *MYC* translocation, there was a positive correlation between *BCL2* translocation and *TP53* mutation (*p* = 0.048), and a negative correlation between *BCL2* and *BCL6* translocation (*p* = 0.034) (supplementary material Table S2).

Among the 234 cases of DLBCL investigated, 153 had data on COO‐classification by Illumina WG‐DASL array from a previous study and 140 of these cases were *MYC* translocation negative 4. We, thus, correlated COO subtypes with genetic abnormalities in cases without *MYC* translocation. As expected 33, *BCL2* translocation was significantly associated with GCB‐DLBCL (*p =* 0.004), although no significant association was seen between *BCL6* translocation and COO subtype (*p* = 0.079, supplementary material Table S2). There was no correlation between *TP53* mutation and COO molecular subtype.

### Distinct impact of *TP53* mutations, *BCL2* and *BCL6* translocations on prognosis of *MYC* translocation positive DLBCL

Of the 81 *MYC* translocation positive DLBCLs investigated, 60 (74.1%) had at least one additional genetic abnormality, namely *TP53* mutation, *BCL2* or *BCL6* translocation. Of these 60 cases, 18 had three abnormalities, including nine cases with *MYC/BCL2* double translocation and *TP53* mutation, four cases with *MYC*/*BCL6* double translocation and *TP53* mutation, and a further five cases with triple translocations (Figure 1A). The remaining 42 cases with a single additional genetic abnormality included 22 with *BCL2* translocation, 14 with *TP53* mutation, and six with *BCL6* translocation (Figure 1A).

To investigate whether the prognosis of these *MYC* translocation positive DLBCLs was affected by the number and nature of additional genetic abnormalities, we performed a series of survival analyses. These were carried out exclusively on cases treated with R‐CHOP or equivalent regimens with a curative intent, and included 67 cases with *MYC* translocation and 101 cases without *MYC* translocation.

We first focused on the *MYC* translocation positive DLBCL, subdivided according to the number (1 or 2) of additional genetic abnormalities regardless of their nature, and examined whether the number of additional genetic abnormalities impacted on patient survival. To our surprise, cases with one, but not those with two additional genetic abnormalities, showed a worse survival than those with an isolated *MYC* translocation. Such difference might be due to an insufficient number of the cases with two additional genetic abnormalities for comparison. Alternatively, the findings suggest that the nature rather than the number of the additional genetic abnormalities may be more important in influencing the prognosis of *MYC* translocation positive DLBCL.

We next examined the survival of patients with *MYC* translocation positive DLBCL purely according to the status of *TP53* mutation, *BCL2* and *BCL6* translocation. The *MYC* translocation positive DLBCL with *TP53* mutation had a significantly worse overall survival than those without *TP53* mutation by multivariate Cox regression analysis adjusted for age (*p* = 0.036, supplementary material Figure S2C). Similarly, *MYC* translocation positive DLBCL with *BCL2* translocation showed a worse survival, albeit not statistically significant, than those lacking the translocation (supplementary material Figure S2A). In contrast, *MYC* translocation positive DLBCL with *BCL6* translocation had a significantly better overall survival than those without *BCL6* translocation by multivariate Cox regression analysis adjusted for age (*p* = 0.035, supplementary material Figure S2B). Despite that the reference subgroup in each of the above analyses comprised of mixed cases that included not only those with isolated *MYC* translocation but also cases with other second hit, the analyses, nonetheless, suggested that *TP53* mutation and possibly *BCL2*, but not *BCL6* translocation may have an adverse effect on patients survival. In view of this and the small number of cases with only *MYC* and *BCL6* translocation, our subsequent analyses focused on *TP53* mutation and *BCL2* translocation.

**Figure 2:**
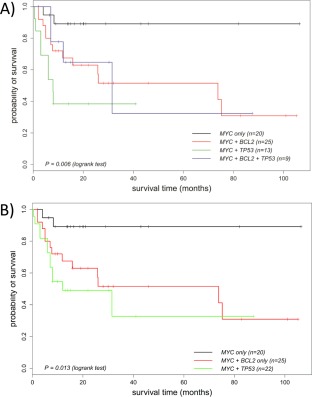
Impact of *TP53* mutation and *BCL2* translocation on overall survival of patients with *MYC* translocation positive DLBCL. (A) *MYC* translocation positive DLBCL are divided into four subgroups: *MYC*/*BCL2* translocation/*TP53* mutation, *MYC* translocation/*TP53* mutation, *MYC*/*BCL2* translocation, and *MYC* translocation only. The cases with the *MYC*/*BCL2* /*TP53* triple‐hit show the worst overall survival, followed by cases with the *MYC/TP53* and those with the *MYC/BCL2* double‐hit. The significant difference in overall survival between cases with the *MYC*/*BCL2* /*TP53* triple‐hit and those with isolated *MYC* translocation is also shown by Cox proportional hazards regression adjusted for age. (B) The cases with *TP53* mutation are combined together irrespective of their *BCL2* translocation status. The cases with *TP53* mutation show a worse overall survival than those with an isolated *MYC* translocation, being statistically significant by Cox regression model adjusted for age (*p* = 0.0059, Table 1). The cases with *BCL2* translocation also show a significantly worse overall survival than those with isolated *MYC* translocation by Cox regression model adjusted for age (*p* = 0.019, Table 1).

We divided *MYC* translocation positive DLBCL into the following subgroups according to *TP53* mutation and *BCL2* translocation status: *MYC/BCL2* double translocation with *TP53* mutation, *MYC* single translocation with *TP53* mutation, *MYC*/*BCL2* double translocation, and isolated *MYC* translocation. In comparison with the subgroup with isolated *MYC* translocation, all other three subgroups had a significantly worse overall survival by logrank test (Figure 2A). Interestingly, the patients with *MYC/BCL2/TP53* triple hit and the cases with *MYC/TP53* double‐hit appeared to be separated from those with the *MYC*/*BCL2* double‐hit (Figure 2A). In view of these findings, we combined all cases with *TP53* mutation irrespective of the *BCL2* translocation status, and these cases had the worst overall survival (Figure 2B, supplementary material Table S3). Further univariate and multivariate Cox regression analyses adjusted for age confirmed the significant worse overall survival of patients with *MYC*/*TP53* (*n* = 22, *p* = 0.0095 and *p* = 0.0059 respectively) and cases with *MYC*/*BCL2* (*n* = 25, *p* = 0.03 and *p* = 0.019, respectively) double‐hit in comparison with those with isolated *MYC* translocation (*n* = 20) Figure 2B, Table 1).

**Table 1:** Impact of *TP53* mutation and *BCL2* translocation on overall survival of patients with *MYC* translocation positive DLBCL by Cox proportional hazards regression.

	**Univariate analysis** a			**Multivariate analysis adjusted for age**		
	HR	95% CI	*P* value	HR	95% CI	*P* value
*MYC + BCL2* translocation(n=25)	5.11	1.15‐22.7	0.03	6.17	1.34‐28.3	0.019
*MYC* translocation + *TP53* mutation (irrespective of *BCL2* translocation status)(n=22)	7.26	1.62‐32.5	0.0095	8.74	1.87–40.8	0.0059

a In comparison with DLBCL with isolated *MYC* translocation; HR: Hazard ratio; CI: confidence interval.

### Prognostic value of *TP53* mutation, *BCL2* and *BCL6* translocation in *MYC* translocation negative DLBCL

All *MYC* translocation negative DLBCLs were retrieved from HMDS, St James's University Hospital, Leeds and Addenbrooke's hospital, Cambridge, and 101 of these cases treated with R‐CHOP had clinical follow up data. As expected, COO‐classification clearly separated these cases into distinct prognostic groups with the ABC‐DLBCL showing a worse overall survival by logrank test (*p* = 0.011, supplementary material Figure S3D). To our surprise, neither isolated *TP53* mutation nor single *BCL2* translocation had a significant impact on overall survival (supplementary material Figure S3A,C). *BCL6* translocation appeared to be associated with a worse overall survival; however, this was not statistically significant by Cox regression analysis after age adjustment (supplementary material Figure S3B).

## Discussion

This is the first study combining chromosome translocations and *TP53* mutation analysis in a large cohort of DLBCL. We have demonstrated that *MYC* translocation positive DLBCL had a significantly higher frequency of *TP53* mutation and *BCL2* translocation 7, and that DLBCL with *MYC* translocation and *TP53* mutation had the worst overall survival, followed by cases with *MYC/BCL2* double‐hits. These findings have significant implications for using these biomarkers in the prognostic evaluation of patients with DLBCL.

By investigating retrospectively a large cohort of *MYC* translocation positive DLBCL, we showed that the prognostic value of *MYC* translocation was critically influenced by the presence of the second hit, essentially its oncogenic cooperating events. The patients with *MYC/TP53* abnormalities irrespective of *BCL2* translocation status had a significantly worse overall survival than those with an isolated *MYC* translocation. The cases with *MYC/BCL2* double translocations but wild type *TP53* showed an intermediate overall survival, appearing better than those with *MYC*/*TP53* abnormalities, but worse than those with an isolated *MYC* translocation (Figure 2B). These findings suggest that *TP53* mutation may have a more adverse impact on prognosis than *BCL2* translocation in *MYC* translocation positive DLBCL. The importance of the second hit in prognosis of *MYC* translocation positive DLBCL is further emphasized by the observation that cases with an isolated *MYC* translocation appeared to have an overall survival similar to those without *MYC* translocation (supplementary material Figure S4). No adverse impact of *MYC* single translocation in DLBCL was also reported in some previous studies 15, 16, 17, but not supported by others 6, 13, 14. However, these previous studies did not investigate *TP53* mutation and it is unknown whether the discrepancy was caused by a variable presence of *TP53* mutation in cases with single *MYC* translocation among these studies. In view of the retrospective nature of the current study, it remains necessary to confirm these findings in a prospective study containing a large cohort of *MYC* translocation positive DLBCL.

Both *TP53* mutation and *BCL2* translocation are known to cooperate with *MYC* translocation in lymphomagenesis by impeding the proapoptotic activities of MYC. In *Eµ‐Myc* transgenic animals, lymphoma development requires the acquisition of additional genetic alterations, which commonly comprise of disruption of the p19^ARF^‐Mdm2‐p53 pathway or overexpression of Bcl2 34, 35. In addition, *Eµ‐Myc* mice with lymphoma showing a loss of p53 function have a significantly worse survival than those with lymphoma overexpressing Bcl2 36. These findings together with our observations in this study indicate that there is a strong selection of genetic events that cooperates with *MYC* translocation during lymphoma development, and these cooperating events are a major determining factor in modulating the prognostic impact of *MYC* translocation in DLBCL.

The prognostic value of *BCL6* translocation in *MYC* translocation positive DLBCL is unclear. The majority of DLBCL with *MYC* and *BCL6* translocation also had *BCL2* translocation or *TP53* mutation, and the number of cases with only *MYC* and *BCL6* translocation is small for assessing the prognostic impact of *BCL6* translocation in *MYC* translocation positive DLBCL. There was no association between *MYC* and *BCL6* translocation in DLBCL. In addition, there is no direct evidence supporting critical oncogenic cooperation between *MYC* and *BCL6* translocation in lymphoma development. Findings from the present and a previous study 37 suggest that *BCL6* translocation appears to be associated with a better prognosis in *MYC* translocation positive DLBCL, although this differs from that observed by Pillai *et al* 38. There are several potential reasons that might account for the discrepancy among these studies, and these include small numbers of cases with *MYC/BCL6* translocation, difference/potential bias in case selection, variations in clinicopathological features (age, stage, IPI) and treatment, and potential differences in the genetic abnormalities associated with *MYC* translocation among these studies. Therefore, it is important to investigate the prognostic value of *BCL6* translocation in *MYC* translocation positive DLBCL in a prospective study.

The term ‘double‐hit’ lymphoma was originally used to describe aggressive DLBCL with *MYC* and *BCL2* translocation, then subsequently extended to include those with *MYC* and *BCL6* translocation. In light of the findings in this study and the discussion above, the term ‘double‐hit’ lymphoma should be extended to include those with *MYC* translocation and *TP53* mutation. Our data clearly highlight the prognostic significance of *TP53* mutation, even more so than *BCL2* translocation, in *MYC* translocation positive DLBCL. It is therefore pivotal to confirm these findings in a prospective study and investigate *TP53* mutation status in routine clinical practice for cases of *MYC* translocation positive DLBCL, in addition to the current standard investigation for *BCL2* translocation.

*MYC* and *BCL2* translocations are commonly investigated by interphase FISH, while *TP53* mutation can be readily screened for by PCR and Sanger sequencing, or a next generation sequencing‐based approach. Alternatively, DLBCL with *MYC/BCL2* or *MYC/TP53* double‐hit might be screened by combined immunohistochemistry for MYC, BCL2 and TP53 15, 16, 23, 31, 32. The challenges for such an immunohistochemistry‐based approach are reproducibility, accurate assessment of the extent and percentage of staining positivity, and the identification of the best cut‐off value for dichotomy between positive and negative staining results. In addition, combined MYC and BCL2 immunohistochemistry also identifies ∼20% of DLBCL that show concurrent MYC and BCL2 protein expression, but no evidence of their involvement in translocation. This group of DLBCL appears to show an overall survival better than the cases with *MYC*/*BCL2* translocation, but worse than those without these translocations 15, 16, 32, and thus, should be regarded as a separate prognostic group. In contrast, combined MYC and TP53 immunohistochemistry would under‐detect DLBCL with *MYC* translocation and *TP53* mutation, as a high proportion (∼17%) of *TP53* mutations such as frameshift and nonsense mutations result in a truncated protein product that is unlikely to be detectable by immunohistochemistry 23.

Our findings also highlight the importance of separating *MYC* translocation positive DLBCL from negative cases in biomarker validation, particularly the genetic events associated with *MYC* translocation, to avoid the confounding effect of *MYC* translocation. *TP53* mutations have been shown to be significantly associated with poor overall survival in both ABC and GCB‐DLBCL treated with R‐CHOP 22, 23. In line with this, we also found a significant association of *TP53* mutation with poor overall survival in DLBCL when *MYC* translocation positive cases were included (based on unselected cases from HMDS, Leeds /Addenbrooke's Hospital). However, this significant association disappeared after excluding the *MYC* translocation positive cases, indicating a confounding effect of the translocation. Nonetheless, the number of *MYC* translocation negative cases investigated in this study is relatively small and the prognostic value of *TP53* mutation in *MYC* translocation negative DLBCL remains to be elucidated.

In summary, we have shown that *MYC* translocation positive DLBCL has a significantly higher frequency of *TP53* mutation and *BCL2* translocation, and that the cases with *MYC* translocation and *TP53* mutation had the worst overall survival, followed by cases with *MYC/BCL2* double‐hits. It is critical to investigate both *TP53* mutation and *BCL2* rearrangement in *MYC* translocation positive DLBCL, and to distinguish double‐hit DLBCLs from those with an isolated *MYC* translocation in routine clinical practice.

## Contract/grant details

The research in MQD lab was supported by grants from Leukaemia & Lymphoma Research, U.K. Kay. Kendall Leukaemia Fund (KKLF), The Pathological Society of UK & Ireland and the Addenbrooke's Charitable Trust (ACT). NG was supported by a KKLF and an ACT fellowship. Research in JG's lab was funded by Tenovus Tayside; JB was supported by a research grant from the Paul Abrahams Fund.

## Author contributions

AC, SB, NZ, HL, SK, MW & YH collected and analyzed laboratory data; SC performed statistical analyses; NFG collected clinical data; LW assisted with sample preparation; JG, JB, MN, PF, BW, JWG, PW, HED, GAF, ER, PWMJ, AJW and AJ provided cases, diagnosis and clinical follow up data. MQD supervised and coordinated the study and wrote the manuscript. All authors have commented on the manuscript and approved its final version for submission. The authors declare no conflict of interest.

## Supporting information


**Figure S1**. Nature and distribution of *TP53* mutations in primary DLBCL with and without *MYC* translocation. All mutations are reported in the COSMIC somatic mutation database, with the exception of c.672+1G>T, c.783‐1G>A and R333C. There is no apparent difference in the nature and distribution of *TP53* mutation found in primary DLBCL with and without *MYC* translocation. trans+ve: translocation positive; trans‐ve: translocation negative; Mutations seen in the same case are indicated by the same colour scheme with the exception of those in black.

**Figure S2**. Impact of *TP53* mutation, *BCL2* and *BCL6* translocation on overall survival of patients with *MYC* translocation positive DLBCL. trans+ve: translocation positive; trans‐ve: translocation negative.

**Figure S3**. Impact of *TP53* mutation, *BCL2* and *BCL6* translocation, and COO molecular subtype on the overall survival of patients with *MYC* translocation negative DLBCL. These cases are from the Haematological Malignancy Diagnostic Service (HMDS) at St James's University Hospital, Leeds and Addenbrooke's hospital, Cambridge, retrieved based on the availability of lymphoma tissue specimens. All cases included in the survival analysis were treated with R‐CHOP or a rituximab‐containing equivalent regimen. trans+ve: translocation positive; trans‐ve: translocation negative; COO: cell of origin

**Figure S4**. Comparison of overall survival between DLBCL with isolated *MYC* translocation (absence of *TP53* mutation, *BCL2* and *BCL6* translocation) and those without *MYC* translocation. These cases are selected based on the availability of lymphoma tissue specimens from the Haematological Malignancy Diagnostic Service (HMDS) at St James's University Hospital, Leeds, and Addenbrooke's hospital, Cambridge, and all cases included in this figure were treated with R‐CHOP or equivalent regimens. Dotted lines indicate 95% confidence intervals.

Supporting Information Table S1

Supporting Information Table S2

Supporting Information Table S3
