# Supplementary material for: The prognosis of MYC translocation positive diffuse large B‐cell lymphoma depends on the second hit
Source: J Pathol Clin Res. 2015 Mar 30;1(3):125–33. doi: 10.1002/cjp2.10 (PMC4915334; doi:10.1002/cjp2.10)
Supplement: Supplementary file 1 — Figure S1. Nature and distribution of TP53 mutations in primary DLBCL with and without MYC translocation. All mutations are reported in the COSMIC somatic mutation database, with the exception of c.672+1G>T, c.783‐1G>A and R333C. There is no apparent difference in the nature and distribution of TP53 mutation found in primary DLBCL with and without MYC translocation. trans+ve: translocation positive; trans‐ve: translocation negative; Mutations seen in the same case are indicated by the same colour scheme with the exception of those in black. [file CJP2-1-125-s001.pptx]

## Slide 1
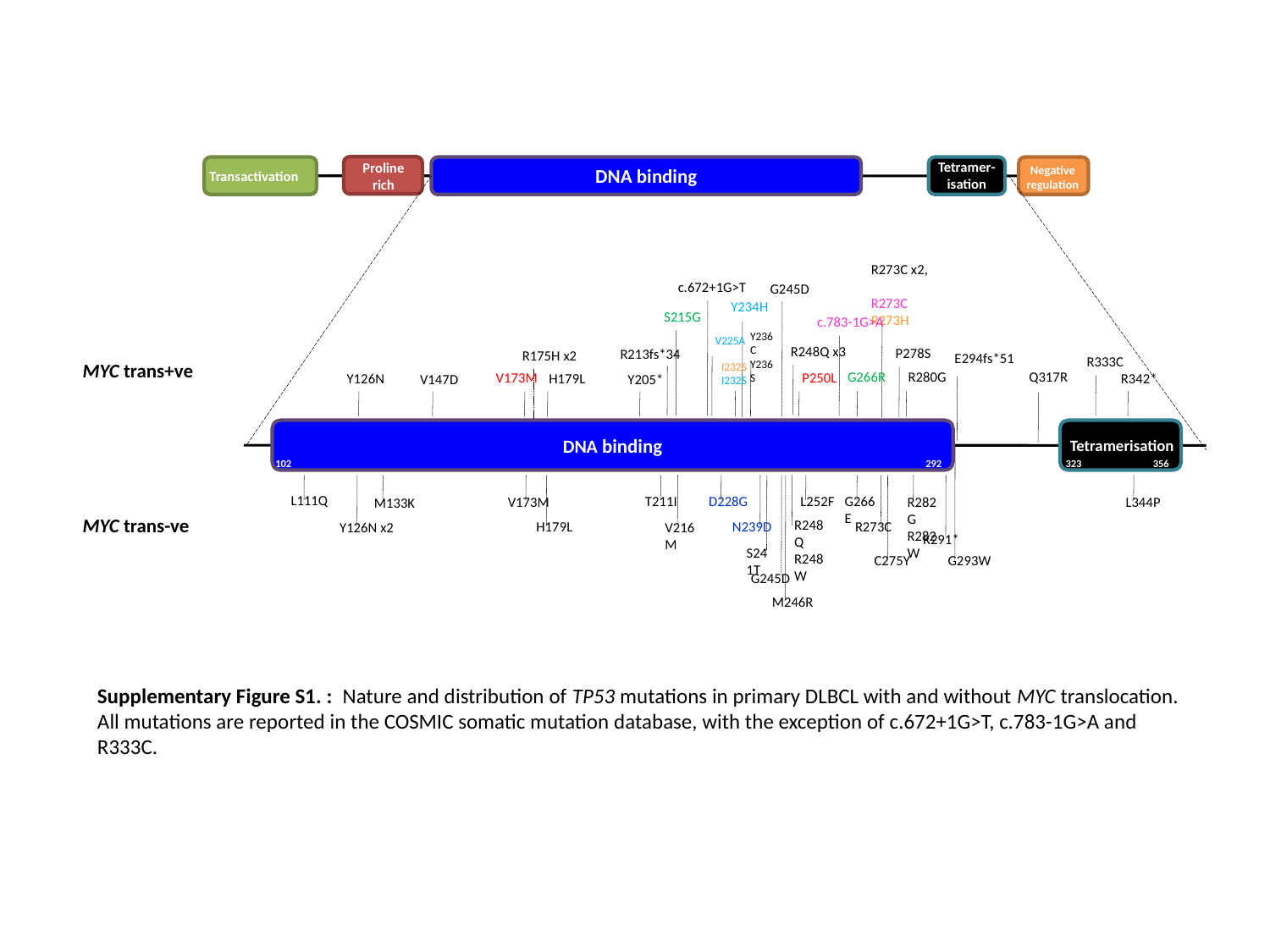

Tetramer-
isation
Proline rich
Negative
regulation
DNA binding
Transactivation
c.672+1G>T
R273C x2,
R273C
R273H
G245D
c.783-1G>A
Y234H
R248Q x3
S215G
R213fs*34
V225A
E294fs*51
R175H x2
P278S
Y236C
Y236S
R333C
V173M
R280G
G266R
Q317R
P250L
R342*
Y126N
H179L
I232S
I232S
V147D
Y205*
MYC trans+ve
DNA binding
Tetramerisation
102
292
323
356
V173M
M133K
L111Q
T211I
L252F
D228G
L344P
G266E
Y126N x2
R282G
R282W
R273C
H179L
N239D
V216M
MYC trans-ve
R248Q
R248W
K291*
G293W
C275Y
S241T
G245D
M246R
Supplementary Figure S1. : Nature and distribution of TP53 mutations in primary DLBCL with and without MYC translocation. All mutations are reported in the COSMIC somatic mutation database, with the exception of c.672+1G>T, c.783-1G>A and R333C.
