# Supplementary material for: The prognosis of MYC translocation positive diffuse large B‐cell lymphoma depends on the second hit
Source: J Pathol Clin Res. 2015 Mar 30;1(3):125–33. doi: 10.1002/cjp2.10 (PMC4915334; doi:10.1002/cjp2.10)
Supplement: Supplementary file 2 — Figure S2. Impact of TP53 mutation, BCL2 and BCL6 translocation on overall survival of patients with MYC translocation positive DLBCL. trans+ve: translocation positive; trans‐ve: translocation negative. [file CJP2-1-125-s002.ppt]

## Slide 1
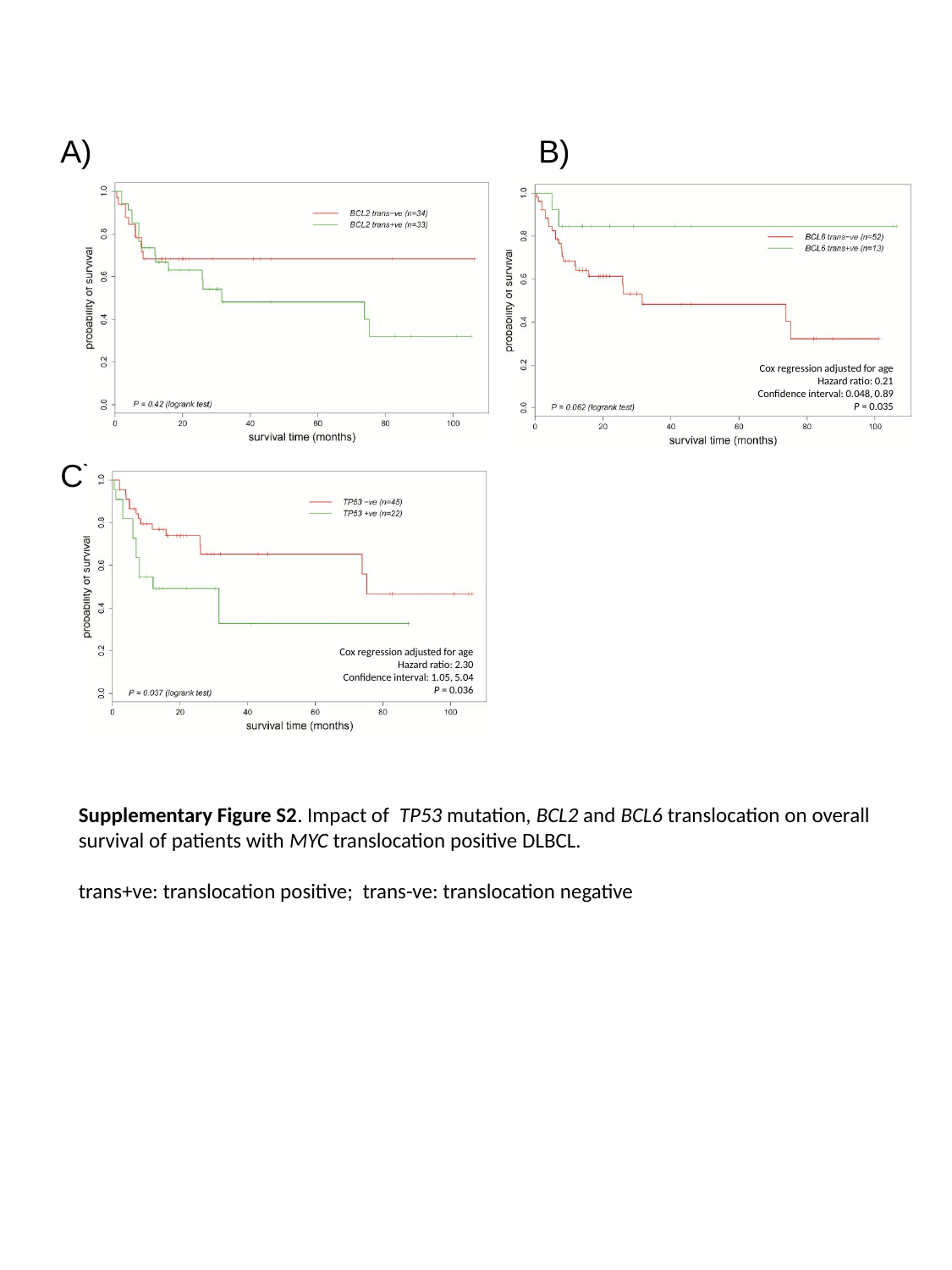

A)
B)
Cox regression adjusted for age
Hazard ratio: 0.21
Confidence interval: 0.048, 0.89
P = 0.035
C)
Cox regression adjusted for age
Hazard ratio: 2.30
Confidence interval: 1.05, 5.04
P = 0.036
Supplementary Figure S2. Impact of TP53 mutation, BCL2 and BCL6 translocation on overall survival of patients with MYC translocation positive DLBCL.
trans+ve: translocation positive; trans-ve: translocation negative
