# Supplementary material for: The prognosis of MYC translocation positive diffuse large B‐cell lymphoma depends on the second hit
Source: J Pathol Clin Res. 2015 Mar 30;1(3):125–33. doi: 10.1002/cjp2.10 (PMC4915334; doi:10.1002/cjp2.10)
Supplement: Supplementary file 3 — Figure S3. Impact of TP53 mutation, BCL2 and BCL6 translocation, and COO molecular subtype on the overall survival of patients with MYC translocation negative DLBCL. These cases are from the Haematological Malignancy Diagnostic Service (HMDS) at St James's University Hospital, Leeds and Addenbrooke's hospital, Cambridge, retrieved based on the availability of lymphoma tissue specimens. All cases included in the survival analysis were treated with R‐CHOP or a rituximab‐containing equivalent regimen. trans+ve: translocation positive; trans‐ve: translocation negative; COO: cell of origin [file CJP2-1-125-s003.ppt]

## Slide 1
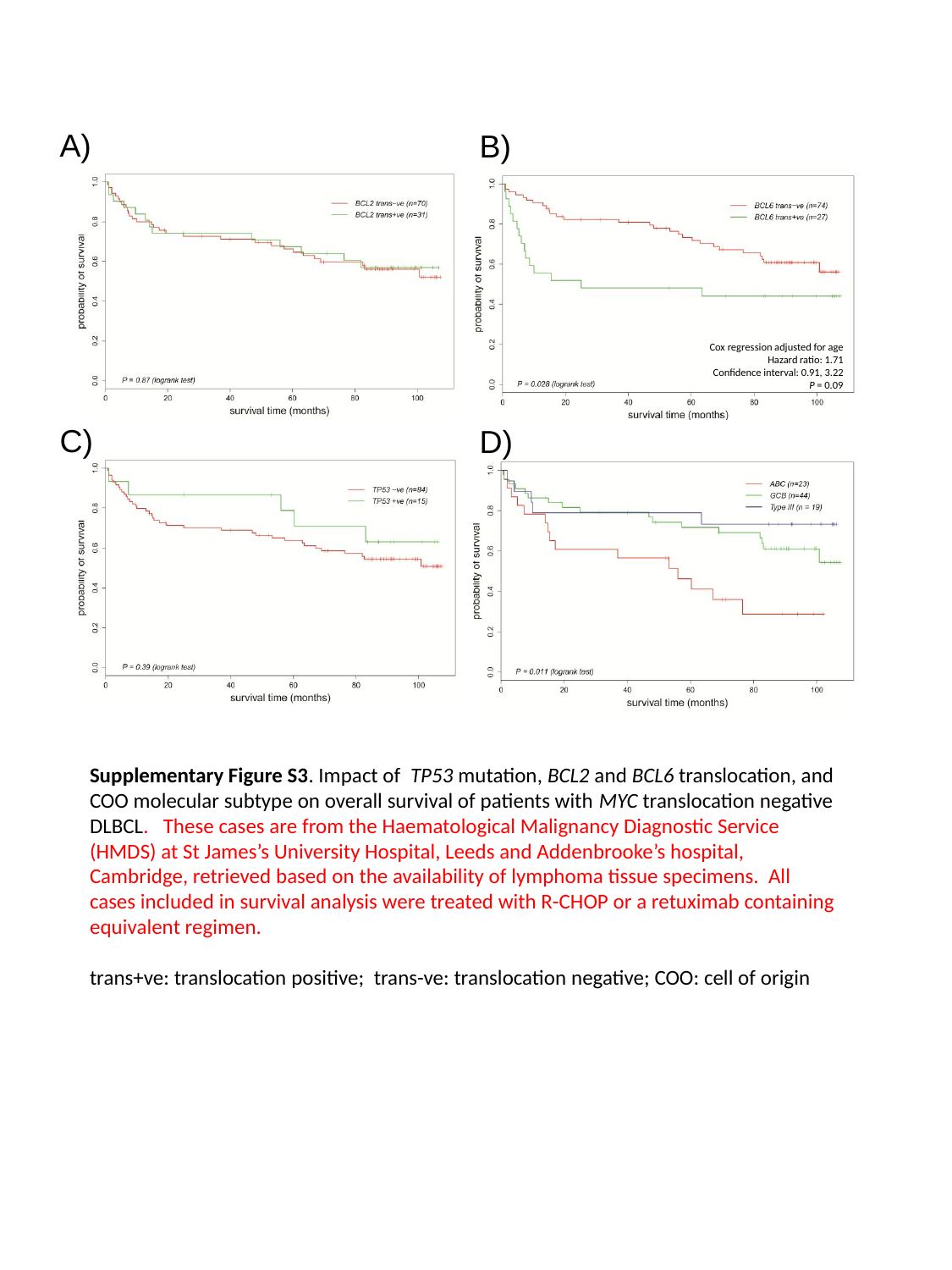

A)
B)
Cox regression adjusted for age
Hazard ratio: 1.71
Confidence interval: 0.91, 3.22
P = 0.09
C)
D)
Cox regression adjusted for age
Hazard ratio: 1.71
Confidence interval: 0.91, 3.22
P = 0.09
Supplementary Figure S3. Impact of TP53 mutation, BCL2 and BCL6 translocation, and COO molecular subtype on overall survival of patients with MYC translocation negative DLBCL. These cases are from the Haematological Malignancy Diagnostic Service (HMDS) at St James’s University Hospital, Leeds and Addenbrooke’s hospital, Cambridge, retrieved based on the availability of lymphoma tissue specimens. All cases included in survival analysis were treated with R-CHOP or a retuximab containing equivalent regimen.
trans+ve: translocation positive; trans-ve: translocation negative; COO: cell of origin
