# Supplementary material for: The prognosis of MYC translocation positive diffuse large B‐cell lymphoma depends on the second hit
Source: J Pathol Clin Res. 2015 Mar 30;1(3):125–33. doi: 10.1002/cjp2.10 (PMC4915334; doi:10.1002/cjp2.10)
Supplement: Supplementary file 4 — Figure S4. Comparison of overall survival between DLBCL with isolated MYC translocation (absence of TP53 mutation, BCL2 and BCL6 translocation) and those without MYC translocation. These cases are selected based on the availability of lymphoma tissue specimens from the Haematological Malignancy Diagnostic Service (HMDS) at St James's University Hospital, Leeds, and Addenbrooke's hospital, Cambridge, and all cases included in this figure were treated with R‐CHOP or equivalent regimens. Dotted lines indicate 95% confidence intervals. [file CJP2-1-125-s004.ppt]

## Slide 1
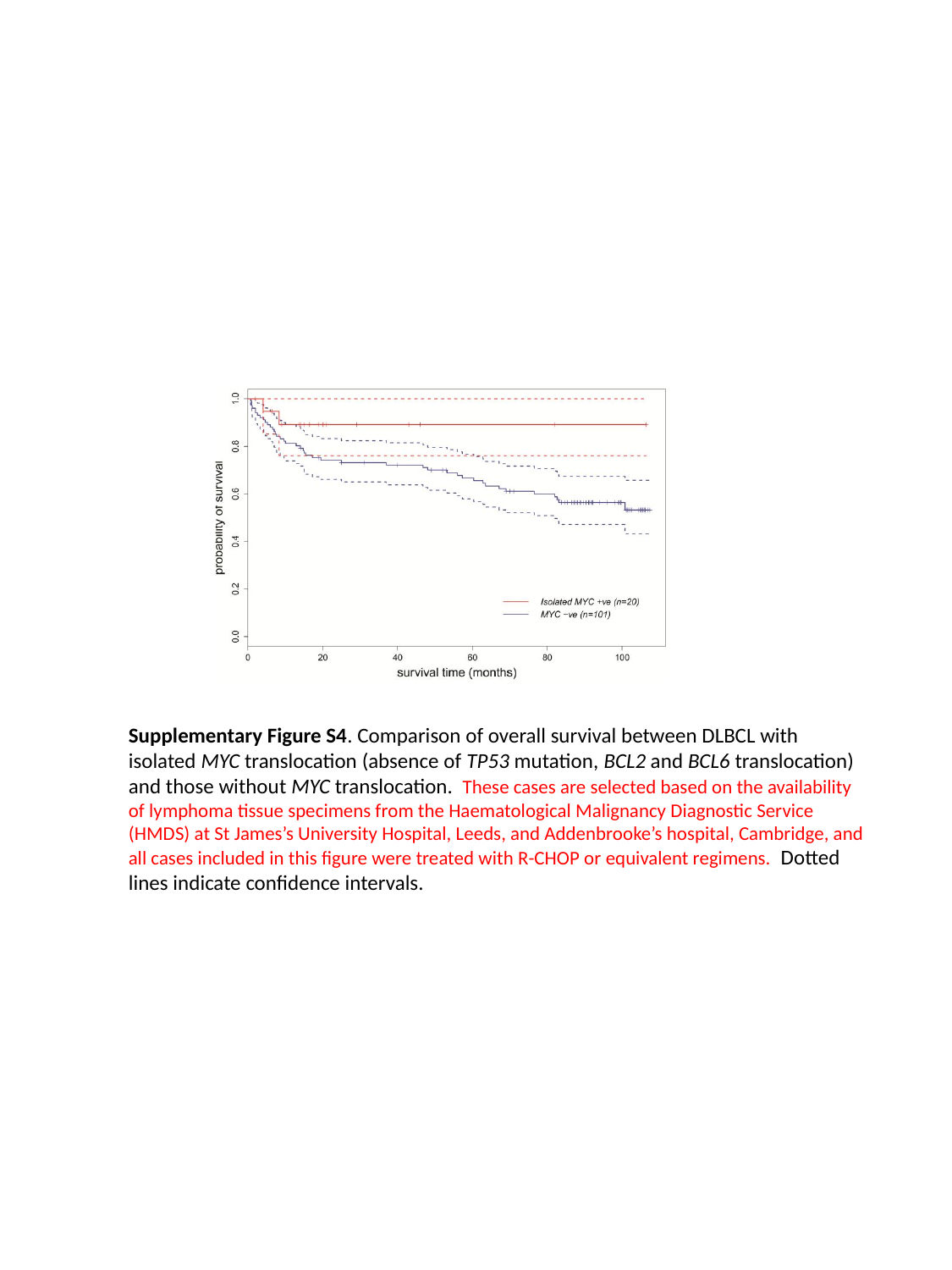

Supplementary Figure S4. Comparison of overall survival between DLBCL with isolated MYC translocation (absence of TP53 mutation, BCL2 and BCL6 translocation) and those without MYC translocation. These cases are selected based on the availability of lymphoma tissue specimens from the Haematological Malignancy Diagnostic Service (HMDS) at St James’s University Hospital, Leeds, and Addenbrooke’s hospital, Cambridge, and all cases included in this figure were treated with R-CHOP or equivalent regimens. Dotted lines indicate confidence intervals.
